# Supplementary figures and images for: Intra-individual variation of particles in exhaled air and of the contents of Surfactant protein A and albumin
Source: PLoS One. 2020 Jan 24;15(1):e0227980. doi: 10.1371/journal.pone.0227980 (PMC6980535; doi:10.1371/journal.pone.0227980)

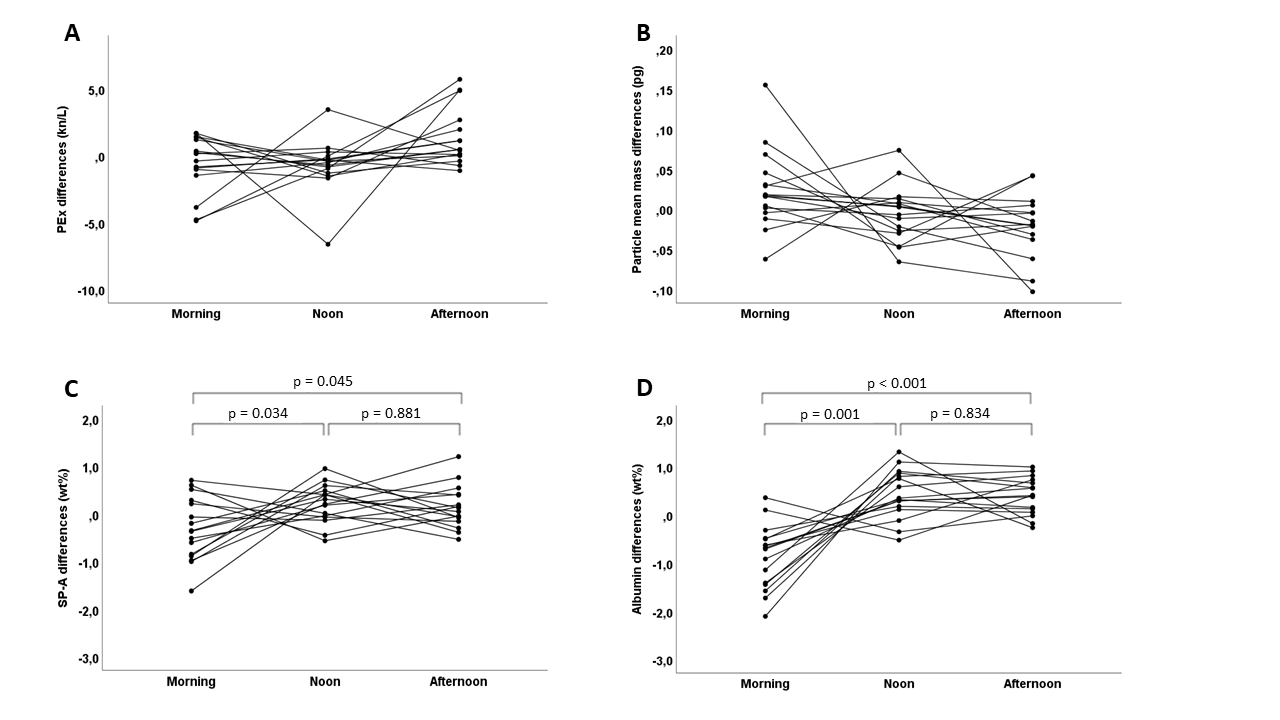

Supplement: S1 Fig — Each measurement was centered around each individual’s mean (yijk* = yijk−yi¯, where i denotes an individual, j a day, k time of the day and yi¯ the mean of all individual’s i measurements) and the mean value of the three days for each individual is presented for each time point (morning, noon and afternoon). For SP-A (C) and albumin (D), p-values, calculated with two-way ANOVA for repeated measures for absolute levels of SP-A and albumin, are given. (TIF) [file pone.0227980.s001.tif]

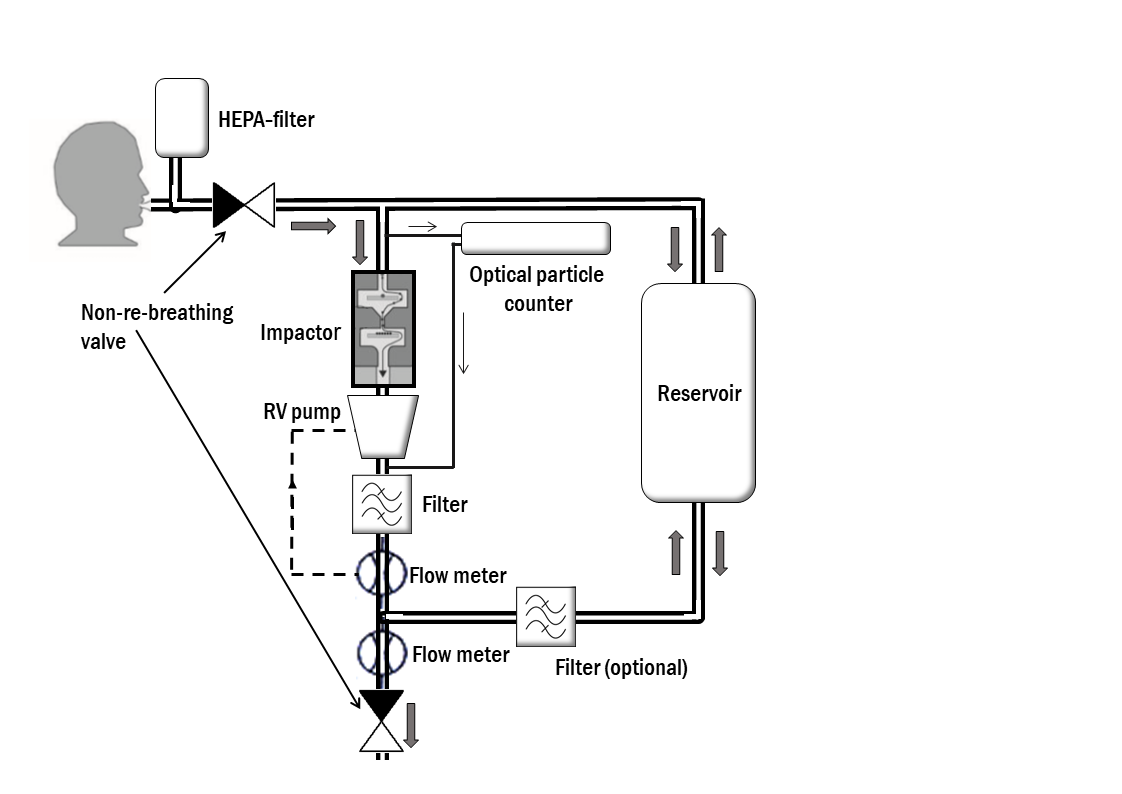

Supplement: S2 Fig — Particle-free air is inhaled through a HEPA-filter. The subject breaths through a mouthpiece via a two-way, non-re-breathing valve into the instrument. A fraction of the air is characterized by an optical particle counter that operates at flow of 20 mL·s-1, while the remainder is drawn through the impactor. Particles are sampled from the aerosol with a two stage inertial impactor that is set up with a constant volumetric flow of 230 mL·s-1 (measured using an ultrasonic flow meter) using a RV pump (rotary vane pump). To handle exhalations exceeding the flow rate through the impactor, a reservoir that can buffer the exhaled air is used. A flow meter also measures the exhalation flow into the reservoir. All parts of the PExA instrument, except the mouthpiece, are in a thermostated box at 36°C. (TIF) [file pone.0227980.s002.tif]
