# Supplementary material for: Intra-individual variation of particles in exhaled air and of the contents of Surfactant protein A and albumin
Source: PLoS One. 2020 Jan 24;15(1):e0227980. doi: 10.1371/journal.pone.0227980 (PMC6980535; doi:10.1371/journal.pone.0227980)
Supplement: S1 File — (PDF) [file pone.0227980.s003.pdf]

| id | alder | sex | datum | tid | Nr_breath | Raw_vol_liter | Corr_vol_liter | Pex_tot_n | Pex_sampl_n |
|---|---|---|---|---|---|---|---|---|---|
| 1 | 65 | 0 | 180314 | 9.15 | 5 | 9,129755 | 9,081974 | 201849,9 | 178228,4 |
| 1 | 65 | 0 | 180314 | 12.30 | 8 | 12,44219 | 12,37823 | 202335,3 | 180145,2 |
| 1 | 65 | 0 | 180314 | 16.00 | 7 | 13,55414 | 13,51208 | 267244,9 | 233223,4 |
| 1 | 65 | 0 | 180416 | 9.00 | 6 | 10,7762 | 10,72373 | 182229,5 | 162090,7 |
| 1 | 65 | 0 | 180416 | 12.30 | 7 | 12,25191 | 12,2005 | 235117,9 | 205381,5 |
| 1 | 65 | 0 | 180416 | 16.30 | 7 | 12,9584 | 12,91558 | 271664,3 | 235351,7 |
| 1 | 65 | 0 | 180502 | 9.20 | 8 | 16,10967 | 15,98153 | 181754,3 | 161834,9 |
| 1 | 65 | 0 | 180502 | 13.00 | 8 | 14,43719 | 14,33237 | 243932,6 | 211544,3 |
| 1 | 65 | 0 | 180502 | 16.15 | 11 | 18,79545 | 18,63489 | 209299,8 | 185653 |
| 1 | 65 | 0 | 180507 | 13.30 | 9 | 15,99576 | 15,89729 | 267660,6 | 235507,6 |
| 1 | 65 | 0 | 180514 | 10.30 | 13 | 22,89293 | 22,77742 | 168458,4 | 149891,8 |
| 2 | 68 | 1 | 180319 | 9.00 | 4 | 18,12861 | 18,10322 | 347928,4 | 295513,3 |
| 2 | 68 | 1 | 180319 | 12.10 | 6 | 28,16647 | 28,12583 | 300554,6 | 250762,9 |
| 2 | 68 | 1 | 180319 | 16.00 | 4 | 18,56415 | 18,532 | 306112,6 | 253790,3 |
| 2 | 68 | 1 | 180419 | 8.15 | 4 | 19,23827 | 19,17967 | 430255,9 | 360628 |
| 2 | 68 | 1 | 180419 | 13.30 | 4 | 20,3795 | 20,34055 | 453748,7 | 376130,6 |
| 2 | 68 | 1 | 180419 | 16.00 | 4 | 20,16871 | 20,14225 | 374199,1 | 309351,6 |
| 2 | 68 | 1 | 180828 | 9.00 | 7 | 31,72474 | 31,6688 | 277288,6 | 229576,5 |
| 2 | 68 | 1 | 180828 | 13.00 | 4 | 18,57043 | 18,52909 | 351682,4 | 289013,6 |
| 2 | 68 | 1 | 180828 | 16.00 | 4 | 17,58275 | 17,53189 | 371046,3 | 282059,2 |
| 2 | 68 | 1 | 180717 | 13.40 | 4 | 18,75652 | 18,7236 | 339171,9 | 273330,5 |
| 2 | 68 | 1 | 180823 | 13.00 | 4 | 17,96354 | 17,9251 | 373945,2 | 312517,1 |
| 3 | 46 | 0 | 180320 | 9.00 | 25 | 85,37041 | 85,1464 | 258529 | 219297,2 |
| 3 | 46 | 0 | 180320 | 12.30 | 29 | 103,327 | 103,0803 | 403181,4 | 326194,9 |
| 3 | 46 | 0 | 180320 | 16.00 | 53 | 173,5826 | 173,1632 | 317463,9 | 259868,1 |
| 3 | 46 | 0 | 180411 | 9.00 | 9 | 31,75509 | 31,65825 | 298861,8 | 253006,2 |
| 3 | 46 | 0 | 180411 | 12.30 | 16 | 55,74582 | 55,59208 | 363013,4 | 297278,4 |
| 3 | 46 | 0 | 180411 | 16.00 | 13 | 49,31574 | 49,20331 | 385971,9 | 310872 |
| 3 | 46 | 0 | 180502 | 9.00 | 10 | 36,00131 | 35,79669 | 310439 | 254394,7 |
| 3 | 46 | 0 | 180502 | 12.30 | 13 | 47,90479 | 47,6574 | 376891,1 | 301878,1 |
| 3 | 46 | 0 | 180502 | 16.00 | 8 | 29,55135 | 29,37103 | 357964,1 | 291966 |
| 3 | 46 | 0 | 180522 | 13.00 | 10 | 35,98858 | 35,91733 | 398481,7 | 321423,4 |
| 3 | 46 | 0 | 180612 | 9.00 | 8 | 28,49704 | 28,40894 | 356369,7 | 292966,4 |
| 4 | 22 | 0 | 180320 | 9.30 | 6 | 24,03555 | 23,98356 | 468186,4 | 378230,8 |
| 4 | 22 | 0 | 180320 | 13.00 | 8 | 36,3583 | 36,27784 | 640117,5 | 485322,1 |
| 4 | 22 | 0 | 180320 | 16.30 | 8 | 34,58079 | 34,50717 | 597546,2 | 453668,8 |
| 4 | 22 | 0 | 180411 | 9.30 | 7 | 24,42283 | 24,36421 | 361831 | 297819,6 |
| 4 | 22 | 0 | 180411 | 13.00 | 9 | 35,48769 | 35,40017 | 466723,4 | 364244,9 |
| 4 | 22 | 0 | 180411 | 16.30 | 8 | 31,2833 | 31,21345 | 496693,5 | 388214,2 |
| 4 | 22 | 0 | 180424 | 9.30 | 6 | 23,87367 | 23,82255 | 380203,3 | 316230,8 |
| 4 | 22 | 0 | 180424 | 12.30 | 8 | 29,3089 | 29,24197 | 466346,3 | 365646,3 |
| 4 | 22 | 0 | 180424 | 15.00 | 7 | 25,33986 | 25,28436 | 441048,8 | 347398,7 |
| 4 | 22 | 0 | 180503 | 13.30 | 6 | 21,14679 | 21,03827 | 434088,9 | 346028,2 |
| 4 | 22 | 0 | 180517 | 9.00 | 7 | 28,10135 | 27,93698 | 501591,2 | 388200,5 |
| 5 | 68 | 0 | 180410 | 9.00 | 4 | 8,126134 | 8,061907 | 388736,5 | 339952,3 |
| 5 | 68 | 0 | 180410 | 13.00 | 4 | 7,418503 | 7,378642 | 303433,2 | 263661,4 |
| 5 | 68 | 0 | 180410 | 16.00 | 4 | 6,373559 | 6,337216 | 230011,2 | 208782,7 |
| 5 | 68 | 0 | 180424 | 9.00 | 4 | 6,725701 | 6,687381 | 219184,9 | 199459,8 |
| 5 | 68 | 0 | 180424 | 13.00 | 4 | 6,352539 | 6,304998 | 218301,9 | 195119,5 |
| 5 | 68 | 0 | 180424 | 15.30 | 4 | 7,294508 | 7,252728 | 360126,8 | 321768,5 |
| 5 | 68 | 0 | 180516 | 9.00 | 4 | 7,398737 | 7,336079 | 284815,9 | 234547,1 |
| 5 | 68 | 0 | 180516 | 13.00 | 6 | 8,306141 | 8,231002 | 157872,7 | 138427,1 |
| 5 | 68 | 0 | 180516 | 16.00 | 4 | 6,481613 | 6,403573 | 279097,8 | 247181,2 |

| | | | | | | | | | |
|---|---|---|---|---|---|---|---|---|---|
| 5 | 68 | 0 | 180521 | 9.00 | 4 | 6,459329 | 6,40249 | 159857,6 | 144668,3 |
| 5 | 68 | 0 | 180528 | 9.30 | 4 | 6,100228 | 6,062505 | 102849,6 | 93839,9 |
| 6 | 61 | 0 | 180503 | 8.30 | 19 | 47,76924 | 47,43092 | 252644,5 | 214171,8 |
| 6 | 61 | 0 | 180503 | 11.10 | 14 | 34,78041 | 34,51564 | 182653,8 | 158441,3 |
| 6 | 61 | 0 | 180503 | 14.30 | 10 | 27,4022 | 27,19378 | 269836,3 | 225291,7 |
| 6 | 61 | 0 | 180521 | 9.30 | 6 | 17,56468 | 17,49128 | 181364,4 | 156004,2 |
| 6 | 61 | 0 | 180521 | 13.30 | 9 | 26,27382 | 26,15951 | 307007,2 | 254850,9 |
| 6 | 61 | 0 | 180521 | 16.15 | 9 | 27,24827 | 27,15218 | 307199,4 | 253538,9 |
| 6 | 61 | 0 | 180612 | 9.30 | 11 | 33,6419 | 33,52462 | 305784,9 | 253603,8 |
| 6 | 61 | 0 | 180612 | 13.30 | 10 | 26,81622 | 26,72513 | 263566,2 | 221084,5 |
| 6 | 61 | 0 | 180612 | 16.00 | 10 | 26,45922 | 26,3796 | 256487,7 | 219146,3 |
| 6 | 61 | 0 | 180418 | 9.00 | 14 | 34,47754 | 34,35615 | 238361,9 | 203527,8 |
| 6 | 61 | 0 | 180620 | 15.00 | 10 | 29,80135 | 29,72303 | 269529,8 | 228611,7 |
| 7 | 37 | 0 | 180522 | 8.00 | 12 | 34,66493 | 34,55544 | 252184,9 | 214215,9 |
| 7 | 37 | 0 | 180522 | 12.45 | 9 | 22,37113 | 22,29612 | 220922,4 | 186706,1 |
| 7 | 37 | 0 | 180522 | 15.00 | 11 | 30,48865 | 30,40111 | 306361 | 253362,8 |
| 7 | 37 | 0 | 180612 | 8.00 | 5 | 15,04498 | 14,99282 | 303764,3 | 247237,4 |
| 7 | 37 | 0 | 180612 | 12.15 | 4 | 12,13582 | 12,09213 | 358450,5 | 294052,8 |
| 7 | 37 | 0 | 180612 | 14.30 | 4 | 11,95131 | 11,91436 | 523735,3 | 431928,6 |
| 7 | 37 | 0 | 180619 | 8.00 | 7 | 20,85645 | 20,80198 | 285129,5 | 234409,8 |
| 7 | 37 | 0 | 180619 | 12.15 | 8 | 24,05885 | 24,00747 | 317477,2 | 258179,5 |
| 7 | 37 | 0 | 180619 | 14.30 | 6 | 17,67322 | 17,62208 | 329839,1 | 266147,1 |
| 7 | 37 | 0 | 180507 | 13.00 | 5,5 | 18,27636 | 18,16373 | 377557,3 | 306116 |
| 7 | 37 | 0 | 180608 | 8.00 | 6 | 17,53065 | 17,46921 | 316379,8 | 259947,1 |
| 8 | 45 | 1 | 180514 | 8.30 | 6 | 28,4598 | 28,35822 | 460262,7 | 358650 |
| 8 | 45 | 1 | 180514 | 12.30 | 6 | 29,00216 | 28,93844 | 492568,9 | 379773,8 |
| 8 | 45 | 1 | 180514 | 16.00 | 6 | 28,53886 | 28,45756 | 435539,1 | 336542,9 |
| 8 | 45 | 1 | 180528 | 9.30 | 4 | 19,80393 | 19,76923 | 475967 | 387614,7 |
| 8 | 45 | 1 | 180528 | 12.30 | 5 | 25,38084 | 25,30569 | 515118,1 | 404020,4 |
| 8 | 45 | 1 | 180528 | 15.30 | 4 | 20,19379 | 20,15867 | 451363,6 | 352158,5 |
| 8 | 45 | 1 | 180611 | 9.30 | 5 | 24,55338 | 24,50431 | 566534,1 | 429089,1 |
| 8 | 45 | 1 | 180611 | 12.30 | 6 | 29,39521 | 29,33769 | 502370 | 390252,5 |
| 8 | 45 | 1 | 180611 | 15.30 | 5 | 25,02777 | 24,97283 | 487018 | 379575,5 |
| 8 | 45 | 1 | 180507 | 16.00 | 6 | 28,4598 | 28,35822 | 460262,7 | 358650 |
| 8 | 45 | 1 | 180521 | 16.00 | 5 | 24,99215 | 24,93918 | 558702,7 | 435292,4 |
| 9 | 41 | 1 | 180515 | 9.30 | 11 | 63,53486 | 63,33369 | 490789,9 | 372906,6 |
| 9 | 41 | 1 | 180515 | 13.00 | 9 | 50,41986 | 50,30556 | 423048,1 | 331867,8 |
| 9 | 41 | 1 | 180515 | 16.30 | 9 | 50,70114 | 50,53934 | 446839,6 | 350956,8 |
| 9 | 41 | 1 | 180530 | 10.00 | 5 | 29,40404 | 29,30141 | 415715,5 | 334151,5 |
| 9 | 41 | 1 | 180530 | 13.00 | 9 | 48,58794 | 48,4856 | 445359,4 | 349772,1 |
| 9 | 41 | 1 | 180530 | 16.00 | 6 | 31,92497 | 31,86537 | 412751 | 331241,2 |
| 9 | 41 | 1 | 180611 | 9.45 | 6 | 32,58944 | 32,51552 | 340853 | 278449,4 |
| 9 | 41 | 1 | 180611 | 13.00 | 13 | 67,94825 | 67,80082 | 415729,8 | 330564,1 |
| 9 | 41 | 1 | 180611 | 16.00 | 12 | 62,46902 | 62,34399 | 427455,6 | 341625 |
| 9 | 41 | 1 | 180621 | 10.00 | 4 | 19,94403 | 19,89744 | 305563,9 | 255114,7 |
| 9 | 41 | 1 | 180627 | 13.30 | 5 | 22,35999 | 22,31253 | 379513,6 | 305521,8 |
| 10 | 66 | 1 | 180614 | 9.00 | 6 | 18,5576 | 18,50368 | 181851,8 | 162391 |
| 10 | 66 | 1 | 180614 | 12.30 | 4 | 13,27916 | 13,23654 | 226784 | 200307,5 |
| 10 | 66 | 1 | 180614 | 15.20 | 5 | 19,6681 | 19,6186 | 273768,1 | 226344,4 |
| 10 | 66 | 1 | 180619 | 9.00 | 6 | 19,48278 | 19,42366 | 230552,8 | 199955,1 |
| 10 | 66 | 1 | 180619 | 13.00 | 4 | 14,43935 | 14,40411 | 258494,5 | 221475,1 |
| 10 | 66 | 1 | 180619 | 15.30 | 5 | 16,55856 | 16,51768 | 210903,6 | 179665,1 |
| 10 | 66 | 1 | 180626 | 9.00 | 7 | 21,67183 | 21,6093 | 203201,6 | 178284,1 |
| 10 | 66 | 1 | 180626 | 12.30 | 4 | 12,98756 | 12,93881 | 232436,5 | 203442,8 |
| 10 | 66 | 1 | 180626 | 16.00 | 4 | 14,09627 | 14,06143 | 241040,9 | 203920,5 |
| 10 | 66 | 1 | 180608 | 8.30 | 8 | 22,2405 | 22,16297 | 194732,8 | 171793,3 |

| | | | | | | | | | |
|---|---|---|---|---|---|---|---|---|---|
| 10 | 66 | 1 | 180717 | 15.20 | 4 | 12,3427 | 12,30531 | 244524,9 | 210875,4 |
| 11 | 49 | 1 | 180718 | 10.15 | 7 | 24,90114 | 24,84516 | 253701,8 | 215967 |
| 11 | 49 | 1 | 180718 | 13.00 | 6 | 23,49338 | 23,42851 | 301507 | 248202,1 |
| 11 | 49 | 1 | 180718 | 17.00 | 5 | 17,52441 | 17,47092 | 304007,2 | 255776,2 |
| 11 | 49 | 1 | 180723 | 10.00 | 4 | 15,49064 | 15,46226 | 291039,4 | 245707,6 |
| 11 | 49 | 1 | 180723 | 12.45 | 6 | 20,9724 | 20,9321 | 361630,3 | 305668,4 |
| 11 | 49 | 1 | 180723 | 16.00 | 4 | 14,01899 | 13,97779 | 438290,6 | 367824,6 |
| 11 | 49 | 1 | 181026 | 8.50 | 4 | 16,30445 | 16,27096 | 342642,7 | 286810,4 |
| 11 | 49 | 1 | 181026 | 12.30 | 6 | 18,4111 | 18,34839 | 330200,5 | 277438,1 |
| 11 | 49 | 1 | 181026 | 15.00 | 6 | 17,33179 | 17,27403 | 214902,2 | 189735,4 |
| 11 | 49 | 1 | 180712 | 10.00 | 5 | 19,59218 | 19,55 | 352347,3 | 295969 |
| 11 | 49 | 1 | 180913 | 11.20 | 4 | 13,94995 | 13,90869 | 276634,7 | 240112,3 |
| 12 | 30 | 0 | 180821 | 9.00 | 17 | 45,80566 | 45,63485 | 292896,3 | 249142,8 |
| 12 | 30 | 0 | 180821 | 12.45 | 11 | 33,59679 | 33,49782 | 316782 | 265542,1 |
| 12 | 30 | 0 | 180821 | 15.15 | 10 | 31,46773 | 31,36831 | 406774,4 | 332941,5 |
| 12 | 30 | 0 | 180905 | 8.00 | 10 | 30,32493 | 30,23335 | 380204,3 | 313398,8 |
| 12 | 30 | 0 | 180905 | 13.00 | 6 | 18,98791 | 18,92481 | 393396,4 | 328416,3 |
| 12 | 30 | 0 | 180905 | 15.30 | 5 | 15,74877 | 15,6993 | 396922 | 325323,1 |
| 12 | 30 | 0 | 180911 | 8.00 | 8 | 23,92951 | 23,8544 | 353754,3 | 294698,3 |
| 12 | 30 | 0 | 180911 | 12.30 | 6 | 19,49611 | 19,4306 | 352092,7 | 290776,4 |
| 12 | 30 | 0 | 180911 | 16.00 | 5 | 15,02729 | 14,96849 | 370986,8 | 309965,6 |
| 12 | 30 | 0 | 180920 | 12.35 | 4 | 15,19745 | 15,15983 | 329203,5 | 273675,1 |
| 12 | 30 | 0 | 180925 | 8.00 | 7 | 23,99101 | 23,92692 | 375295,7 | 302702,5 |
| 13 | 35 | 1 | 181108 | 9.30 | 5 | 32,5564 | 32,46097 | 420007,6 | 327134,8 |
| 13 | 35 | 1 | 181108 | 12.20 | 4 | 24,22874 | 24,15219 | 365091,7 | 291380,8 |
| 13 | 35 | 1 | 181108 | 16.00 | 4 | 25,96294 | 25,88457 | 400369,9 | 319867,8 |
| 13 | 35 | 1 | 180910 | 9.45 | 6 | 39,98106 | 39,89184 | 450561,9 | 345878,3 |
| 13 | 35 | 1 | 180910 | 12.10 | 5 | 33,72364 | 33,6602 | 383749,9 | 300926,3 |
| 13 | 35 | 1 | 180910 | 14.30 | 5 | 31,90764 | 31,83351 | 461668,8 | 372680,1 |
| 13 | 35 | 1 | 180925 | 9.30 | 5 | 30,40669 | 30,33664 | 376845,1 | 296729,1 |
| 13 | 35 | 1 | 180925 | 12.15 | 5 | 33,83798 | 33,76894 | 438846,2 | 344939,5 |
| 13 | 35 | 1 | 180925 | 14.40 | 4 | 26,99236 | 26,92709 | 456777,4 | 361010,4 |
| 13 | 35 | 1 | 180829 | 15.00 | 4 | 26,94036 | 26,8812 | 399838 | 315479,1 |
| 13 | 35 | 1 | 181008 | 15.00 | 4 | 26,75977 | 26,68878 | 376129,9 | 299514,4 |
| 14 | 33 | 1 | 180925 | 12.30 | 8 | 21,4396 | 21,34819 | 241325 | 209668,1 |
| 14 | 33 | 1 | 180925 | 14.20 | 11 | 27,69661 | 27,59145 | 299107,5 | 254232,5 |
| 14 | 33 | 1 | 180925 | 16.45 | 10 | 26,58084 | 26,44375 | 311377,7 | 262871,6 |
| 14 | 33 | 1 | 181002 | 13.30 | 17 | 42,15856 | 41,99802 | 251375,6 | 218140,6 |
| 14 | 33 | 1 | 181002 | 15.20 | 14 | 37,7228 | 37,58931 | 275862,7 | 234261,8 |
| 14 | 33 | 1 | 181002 | 17.00 | 22 | 57,12491 | 56,9148 | 300100,5 | 254754 |
| 14 | 33 | 1 | 181024 | 10.15 | 10 | 31,07588 | 30,93335 | 392160 | 327474,2 |
| 14 | 33 | 1 | 181024 | 12.30 | 8 | 25,39807 | 25,29549 | 401943,7 | 327890,3 |
| 14 | 33 | 1 | 181024 | 15.00 | 6 | 22,60522 | 22,51335 | 499032,1 | 397862,1 |
| 14 | 33 | 1 | 180904 | 13.30 | 15 | 40,54082 | 40,3751 | 371714 | 304974,5 |
| 14 | 33 | 1 | 180910 | 10.45 | 10 | 27,2845 | 27,17178 | 348511,7 | 294705 |
| 15 | 24 | 1 | 180910 | 10.00 | 9 | 29,57065 | 29,48797 | 365175,1 | 297781,9 |
| 15 | 24 | 1 | 180910 | 12.45 | 13 | 38,6403 | 38,5228 | 278032,7 | 236089,9 |
| 15 | 24 | 1 | 180910 | 15.15 | 15 | 42,9577 | 42,78481 | 282867,6 | 235303,8 |
| 15 | 24 | 1 | 181115 | 8.50 | 12 | 54,26375 | 54,11918 | 494580,2 | 381286,4 |
| 15 | 24 | 1 | 181115 | 13.00 | 13 | 58,70138 | 58,53592 | 481103 | 377100,7 |
| 15 | 24 | 1 | 181115 | 16.00 | 10 | 47,51775 | 47,38768 | 434400 | 340684,4 |
| 15 | 24 | 1 | 181005 | 8.30 | 13 | 46,01402 | 45,89205 | 402831 | 317607,9 |
| 15 | 24 | 1 | 181005 | 13.00 | 10 | 41,25212 | 41,13019 | 395918,3 | 313403,5 |
| 15 | 24 | 1 | 181005 | 15.00 | 14 | 58,59595 | 58,43711 | 403708,8 | 315942 |
| 15 | 24 | 1 | 181010 | 16.00 | 11 | 50,52879 | 50,36959 | 433406,9 | 337264,2 |
| 15 | 24 | 1 | 181019 | 13.00 | 13 | 60,00266 | 59,83468 | 469328,1 | 362958,3 |

| | | | | | | | | | |
|---|---|---|---|---|---|---|---|---|---|
| 16 | 42 | 0 | 181003 | 9.00 | 19 | 44,95921 | 44,76137 | 455372 | 355354,6 |
| 16 | 42 | 0 | 181003 | 12.45 | 16 | 38,95614 | 38,79667 | 410807,1 | 326844,9 |
| 16 | 42 | 0 | 181003 | 15.30 | 18 | 43,78045 | 43,58777 | 424938,8 | 337093,1 |
| 16 | 42 | 0 | 181011 | 9.30 | 11 | 25,67231 | 25,53837 | 363114,7 | 288904,1 |
| 16 | 42 | 0 | 181011 | 12.40 | 12 | 28,57777 | 28,4278 | 404670,6 | 321804,9 |
| 16 | 42 | 0 | 181011 | 15.00 | 17 | 41,03343 | 40,83778 | 503309,6 | 384192,3 |
| 16 | 42 | 0 | 181018 | 9.45 | 19 | 46,56224 | 46,3706 | 417297,1 | 341528,1 |
| 16 | 42 | 0 | 181018 | 12.45 | 19 | 48,59974 | 48,38187 | 475390,2 | 374043,8 |
| 16 | 42 | 0 | 181018 | 15.40 | 22 | 56,20859 | 55,97816 | 484540,8 | 378610,8 |
| 16 | 42 | 0 | 180913 | 10.00 | 10 | 23,91706 | 23,80071 | 315154,8 | 262460,7 |
| 16 | 42 | 0 | 180925 | 11.10 | 24 | 53,44119 | 53,18995 | 372491,2 | 295322,2 |

| Pex_tot_ng | Pex_sampl_ng | pg_perparticle | Pex_nperL | Pex_ngperL | SpA_percpex | ALB_percpex | kvot_ALB_SpA |
|---|---|---|---|---|---|---|---|
| 162,6725 | 149,217 | 0,805908 | 22225,34 | 17,91158 | 2,546342 | 4,617392 | 1,813344 |
| 134,6003 | 123,4775 | 0,665234 | 16346,05 | 10,87396 | 4,630636 | 7,707451 | 1,664448 |
| 138,6715 | 126,8307 | 0,518893 | 19778,23 | 10,26279 | 3,408695 | 7,757818 | 2,275891 |
| 159,185 | 146,1215 | 0,873541 | 16993,1 | 14,84417 | 3,285729 | 4,824582 | 1,468344 |
| 135,2541 | 123,788 | 0,575261 | 19271,17 | 11,08595 | 4,619021 | 8,078891 | 1,749048 |
| 147,7436 | 135,0625 | 0,543846 | 21033,84 | 11,43918 | 4,212842 | 8,004029 | 1,899912 |
| 140,8211 | 129,2375 | 0,774788 | 11372,77 | 8,81149 | 2,939994 | 5,257015 | 1,788104 |
| 134,8773 | 123,3263 | 0,552929 | 17019,69 | 9,410677 | 5,025276 | 8,528906 | 1,697202 |
| 137,8607 | 126,4239 | 0,658676 | 11231,61 | 7,397988 | 3,573898 | 8,24304 | 2,306456 |
| 135,7211 | 124,2287 | 0,507064 | 16836,87 | 8,537377 | 5,314752 | 9,226456 | 1,736009 |
| 130,0076 | 119,3061 | 0,771749 | 7395,852 | 5,707739 | 4,555165 | 8,355316 | 1,834251 |
| 224,5904 | 205,1705 | 0,645508 | 19219,14 | 12,4061 | 3,2466 | 4,513097 | 1,390099 |
| 145,556 | 132,3899 | 0,484291 | 10686,07 | 5,175171 | 5,238124 | 8,165613 | 1,558881 |
| 154,8775 | 140,8423 | 0,505949 | 16518,05 | 8,357298 | 6,533164 | 7,629443 | 1,167802 |
| 304,3163 | 277,8903 | 0,707291 | 22432,92 | 15,86661 | 2,439412 | 3,216467 | 1,318542 |
| 249,1925 | 226,8152 | 0,549186 | 22307,59 | 12,25102 | 4,763211 | 7,072719 | 1,484864 |
| 179,5131 | 163,0888 | 0,479726 | 18577,82 | 8,912265 | 5,09855 | 7,007993 | 1,374507 |
| 134,0224 | 121,7918 | 0,483332 | 8755,892 | 4,232001 | 5,117904 | 5,564993 | 1,087358 |
| 171,3358 | 155,588 | 0,487189 | 18980,02 | 9,246855 | 4,351868 | 4,997529 | 1,148364 |
| 154,0528 | 138,2237 | 0,415185 | 21164,08 | 8,787003 | 4,395112 | 4,683423 | 1,065598 |
| 141,4358 | 127,8333 | 0,417003 | 18114,67 | 7,553877 | 5,468737 | 5,750832 | 1,051583 |
| 172,3387 | 156,6893 | 0,460866 | 20861,54 | 9,61438 | 4,14833 | 4,888402 | 1,178402 |
| 131,7161 | 120,0825 | 0,509483 | 3036,288 | 1,546937 | 2,867094 | 4,376701 | 1,526529 |
| 133,3631 | 120,0504 | 0,330777 | 3911,336 | 1,293779 | 5,402562 | 7,231596 | 1,338549 |
| 131,9507 | 119,4917 | 0,41564 | 1833,322 | 0,762002 | 4,949103 | 7,345935 | 1,484296 |
| 136,5414 | 124,321 | 0,456871 | 9440,251 | 4,312981 | 4,921946 | 4,460253 | 0,906197 |
| 133,3158 | 120,4819 | 0,367248 | 6529,948 | 2,398108 | 5,644887 | 6,913104 | 1,224667 |
| 133,898 | 120,5726 | 0,346911 | 7844,429 | 2,721321 | 5,033693 | 6,801677 | 1,35123 |
| 135,4 | 122,7236 | 0,436157 | 8672,283 | 3,782474 | 3,454396 | 4,727522 | 1,368552 |
| 134,7401 | 121,3095 | 0,357504 | 7908,343 | 2,827265 | 4,979728 | 6,997982 | 1,405294 |
| 132,9378 | 120,0948 | 0,371372 | 12187,66 | 4,526155 | 5,449051 | 7,335762 | 1,346246 |
| 135,274 | 121,7819 | 0,339474 | 11094,41 | 3,76626 | 7,495539 | 9,106352 | 1,214903 |
| 142,3143 | 128,8669 | 0,399344 | 12544,28 | 5,009489 | 8,21747 | 5,303819 | 0,645432 |
| 136,3954 | 122,381 | 0,291327 | 19521,14 | 5,687038 | 6,200278 | 4,53096 | 0,730767 |
| 146,98 | 129,0996 | 0,229614 | 17644,86 | 4,051509 | 6,304011 | 6,451638 | 1,023418 |
| 144,4314 | 127,199 | 0,241708 | 17316,58 | 4,185549 | 7,627162 | 6,472518 | 0,848614 |
| 133,1245 | 120,4022 | 0,367919 | 14850,93 | 5,463938 | 6,078914 | 5,510012 | 0,906414 |
| 135,9191 | 121,1957 | 0,29122 | 13184,22 | 3,839504 | 8,113682 | 8,59863 | 1,059769 |
| 138,5295 | 123,3844 | 0,278903 | 15912,8 | 4,438134 | 5,950531 | 7,765476 | 1,305006 |
| 139,8091 | 126,6412 | 0,367722 | 15959,81 | 5,868773 | 4,492985 | 3,997398 | 0,889698 |
| 135,0338 | 120,4846 | 0,289557 | 15947,84 | 4,617807 | 7,03608 | 6,434869 | 0,914553 |
| 138,4162 | 123,8934 | 0,313834 | 17443,54 | 5,474378 | 6,118341 | 6,516027 | 1,064999 |
| 139,3699 | 125,0683 | 0,321063 | 20633,3 | 6,624589 | 7,101277 | 5,892282 | 0,82975 |
| 139,4319 | 123,9475 | 0,277979 | 17954,38 | 4,990944 | 5,643103 | 5,739266 | 1,017041 |
| 227,758 | 208,4922 | 0,585893 | 48218,93 | 28,25114 | 3,340456 | 6,270913 | 1,877263 |
| 161,9615 | 148,0887 | 0,533763 | 41123,18 | 21,95005 | 3,117375 | 9,233188 | 2,961847 |
| 190,1789 | 174,7975 | 0,826825 | 36295,3 | 30,00985 | 4,048665 | 7,994536 | 1,97461 |
| 212,0997 | 195,0022 | 0,967675 | 32775,89 | 31,7164 | 2,616244 | 5,477147 | 2,093515 |
| 146,296 | 134,2543 | 0,670154 | 34623,62 | 23,20317 | 4,233026 | 8,781095 | 2,074425 |
| 271,8755 | 249,5611 | 0,754944 | 49653,99 | 37,48596 | 3,745205 | 6,997144 | 1,868294 |
| 182,693 | 166,4532 | 0,641442 | 38824 | 24,90335 | 4,410864 | 8,941392 | 2,02713 |
| 147,9256 | 135,6887 | 0,936993 | 19180,25 | 17,97176 | 5,282228 | 10,02778 | 1,8984 |
| 215,8718 | 198,0354 | 0,773463 | 43584,69 | 33,71116 | 3,456712 | 8,588882 | 2,484697 |

| | | | | | | | |
|---|---|---|---|---|---|---|---|
| 146,5958 | 134,7262 | 0,91704 | 24968,03 | 22,89668 | 2,843648 | 7,56691 | 2,660987 |
| 149,8773 | 137,8409 | 1,457248 | 16964,86 | 24,72201 | 3,989564 | 5,781535 | 1,449165 |
| 134,6727 | 122,8196 | 0,533052 | 5326,579 | 2,839343 | 3,567483 | 6,625113 | 1,857084 |
| 132,9247 | 121,7233 | 0,727741 | 5291,913 | 3,851144 | 2,941001 | 6,895228 | 2,344517 |
| 134,8563 | 122,711 | 0,499771 | 9922,721 | 4,959086 | 4,540907 | 7,467222 | 1,644434 |
| 141,3243 | 129,3771 | 0,779228 | 10368,85 | 8,0797 | 2,304559 | 4,012502 | 1,741115 |
| 137,0595 | 124,4638 | 0,446437 | 11735,97 | 5,239374 | 4,333397 | 6,640481 | 1,532396 |
| 142,1827 | 129,0892 | 0,462835 | 11313,99 | 5,236512 | 4,484499 | 6,384516 | 1,423685 |
| 134,4769 | 122,0806 | 0,439776 | 9121,204 | 4,011287 | 4,763094 | 6,911108 | 1,45097 |
| 133,496 | 121,5518 | 0,506499 | 9862,11 | 4,995149 | 4,776733 | 6,957281 | 1,456494 |
| 149,8752 | 136,891 | 0,584337 | 9722,958 | 5,681481 | 4,781074 | 6,885293 | 1,440114 |
| 134,9013 | 123,1774 | 0,565951 | 6937,97 | 3,926553 | 2,977413 | 6,216313 | 2,087823 |
| 138,5643 | 126,3352 | 0,514096 | 9068,044 | 4,661848 | 3,50504 | 5,697531 | 1,625525 |
| 132,9896 | 121,2988 | 0,52735 | 7297,979 | 3,848586 | 5,567067 | 6,730286 | 1,208947 |
| 132,9681 | 121,353 | 0,601877 | 9908,558 | 5,963735 | 5,333255 | 8,900335 | 1,668837 |
| 132,7591 | 120,4546 | 0,433342 | 10077,3 | 4,366915 | 7,523972 | 10,35118 | 1,37576 |
| 142,8544 | 129,5215 | 0,47028 | 20260,65 | 9,528186 | 5,248794 | 6,092993 | 1,160837 |
| 149,0972 | 135,0583 | 0,415949 | 29643,29 | 12,3301 | 4,819366 | 6,334084 | 1,314298 |
| 246,7679 | 224,0769 | 0,471169 | 43958,34 | 20,71181 | 5,866579 | 6,211762 | 1,058839 |
| 134,4788 | 122,0711 | 0,471641 | 13706,84 | 6,464712 | 6,727361 | 5,645953 | 0,839252 |
| 138,4455 | 125,3664 | 0,43608 | 13224,1 | 5,766767 | 5,191944 | 6,233134 | 1,20054 |
| 137,385 | 124,1895 | 0,416521 | 18717,38 | 7,796185 | 3,764647 | 5,243237 | 1,392757 |
| 140,5014 | 126,8242 | 0,372133 | 20786,34 | 7,735274 | 3,707499 | 9,011884 | 2,430718 |
| 147,1915 | 133,5769 | 0,465237 | 18110,71 | 8,425769 | 6,059476 | 6,66961 | 1,100691 |
| 137,8069 | 122,9531 | 0,299409 | 16230,31 | 4,859503 | 4,38664 | 4,064836 | 0,92664 |
| 135,4707 | 120,2957 | 0,275029 | 17021,27 | 4,681342 | 7,459508 | 6,072908 | 0,814116 |
| 136,5294 | 121,8156 | 0,313472 | 15304,87 | 4,79765 | 7,609533 | 6,049617 | 0,795005 |
| 164,4403 | 148,3139 | 0,345487 | 24076,15 | 8,317993 | 4,676323 | 3,627502 | 0,775717 |
| 162,1525 | 145,0499 | 0,314787 | 20355,82 | 6,40775 | 6,40458 | 4,580843 | 0,715245 |
| 143,2901 | 128,0979 | 0,31746 | 22390,55 | 7,108113 | 8,284311 | 5,068446 | 0,611813 |
| 146,8571 | 129,6641 | 0,25922 | 23119,77 | 5,993114 | 4,964644 | 4,207132 | 0,847419 |
| 145,2073 | 129,3398 | 0,289045 | 17123,71 | 4,949513 | 6,235248 | 5,034452 | 0,807418 |
| 137,999 | 122,9119 | 0,283355 | 19501,92 | 5,525967 | 6,593238 | 5,189747 | 0,787132 |
| 137,8069 | 122,9531 | 0,299409 | 16230,31 | 4,859503 | 7,833723 | 5,291654 | 0,675497 |
| 153,213 | 136,3039 | 0,27423 | 22402,61 | 6,143465 | 7,37057 | 5,875788 | 0,797196 |
| 136,6642 | 121,0847 | 0,278458 | 7749,27 | 2,157844 | 2,725774 | 8,633273 | 3,167274 |
| 137,1779 | 122,8151 | 0,324261 | 8409,568 | 2,726894 | 3,57818 | 9,598981 | 2,682643 |
| 143,1714 | 128,1637 | 0,320409 | 8841,423 | 2,832871 | 3,48975 | 10,30489 | 2,952903 |
| 151,5269 | 136,5498 | 0,364497 | 14187,56 | 5,171319 | 2,771516 | 6,193124 | 2,234562 |
| 141,8916 | 126,9967 | 0,3186 | 9185,395 | 2,926468 | 3,191198 | 9,045224 | 2,834429 |
| 144,2319 | 129,8276 | 0,34944 | 12952,96 | 4,526289 | 3,199252 | 8,543548 | 2,670483 |
| 140,023 | 126,7447 | 0,410802 | 10482,78 | 4,306344 | 3,04602 | 6,456474 | 2,119643 |
| 136,4984 | 122,5108 | 0,328334 | 6131,633 | 2,013226 | 3,79657 | 8,63372 | 2,274084 |
| 137,3252 | 123,2876 | 0,321262 | 6856,405 | 2,202702 | 3,100504 | 9,131379 | 2,945127 |
| 144,0384 | 130,9785 | 0,471385 | 15356,95 | 7,239041 | 1,720023 | 3,978796 | 2,313223 |
| 141,092 | 127,2261 | 0,371771 | 17008,99 | 6,323445 | 4,295889 | 7,850274 | 1,827392 |
| 145,8344 | 133,8774 | 0,801941 | 9827,874 | 7,881376 | 4,382031 | 5,721356 | 1,30564 |
| 175,9249 | 161,3584 | 0,775738 | 17133,18 | 13,29085 | 3,439563 | 5,878387 | 1,70905 |
| 142,3267 | 129,4322 | 0,51988 | 13954,52 | 7,254681 | 4,353617 | 5,192366 | 1,192656 |
| 137,0535 | 125,3709 | 0,594456 | 11869,69 | 7,056006 | 5,429838 | 6,001875 | 1,105351 |
| 185,15 | 169,3723 | 0,716263 | 17945,89 | 12,85397 | 4,003069 | 4,54515 | 1,135417 |
| 134,3334 | 122,7377 | 0,636942 | 12768,35 | 8,1327 | 4,255223 | 5,258888 | 1,235867 |
| 149,8714 | 137,3698 | 0,73755 | 9403,435 | 6,935504 | 4,531104 | 6,056497 | 1,336649 |
| 180,1857 | 165,1563 | 0,775204 | 17964,29 | 13,92599 | 3,401599 | 5,478162 | 1,610467 |
| 140,2187 | 127,947 | 0,581722 | 17141,99 | 9,971866 | 3,786663 | 8,250896 | 2,178936 |
| 136,8755 | 125,4905 | 0,702889 | 8786,405 | 6,175867 | 5,208264 | 7,036472 | 1,35102 |

| | | | | | | | |
|---|---|---|---|---|---|---|---|
| 182,9581 | 167,4889 | 0,748219 | 19871,49 | 14,86822 | 3,653291 | 5,177986 | 1,417349 |
| 157,3432 | 143,7251 | 0,620189 | 10211,32 | 6,33295 | 3,359671 | 7,688627 | 2,288506 |
| 138,0323 | 125,265 | 0,457808 | 12869,24 | 5,89164 | 3,965336 | 7,684019 | 1,937798 |
| 140,4179 | 127,7714 | 0,46189 | 17400,75 | 8,037235 | 5,129356 | 7,848409 | 1,530096 |
| 167,8978 | 153,1629 | 0,57689 | 18822,56 | 10,85855 | 3,105091 | 5,790934 | 1,86498 |
| 186,444 | 169,9309 | 0,515565 | 17276,35 | 8,907083 | 5,362604 | 8,243334 | 1,537189 |
| 235,598 | 214,6578 | 0,537538 | 31356,22 | 16,85517 | 3,048973 | 6,955506 | 2,281262 |
| 134,6427 | 122,1916 | 0,392954 | 21058,54 | 8,275031 | 4,763226 | 7,010397 | 1,471775 |
| 153,1463 | 139,3375 | 0,463798 | 17996,16 | 8,34658 | 3,9598 | 8,32756 | 2,103026 |
| 151,1525 | 138,589 | 0,703354 | 12440,77 | 8,75027 | 2,749393 | 6,393021 | 2,325248 |
| 175,6159 | 159,9005 | 0,498417 | 18022,88 | 8,98291 | 2,989417 | 5,695411 | 1,905191 |
| 164,4738 | 150,4653 | 0,594552 | 19889,34 | 11,82525 | 4,129079 | 8,300251 | 2,010194 |
| 139,0692 | 126,7426 | 0,474807 | 6418,259 | 3,047433 | 6,095105 | 4,289302 | 0,703729 |
| 136,3284 | 123,8916 | 0,430354 | 9456,796 | 4,069771 | 7,685398 | 4,737029 | 0,616367 |
| 143,3642 | 129,4548 | 0,352442 | 12967,69 | 4,570352 | 8,943601 | 5,17676 | 0,578823 |
| 143,3758 | 129,7551 | 0,377102 | 12575,66 | 4,742306 | 8,075099 | 2,921026 | 0,361733 |
| 156,9578 | 142,4208 | 0,398981 | 20787,34 | 8,293759 | 8,413559 | 3,896213 | 0,463087 |
| 144,6558 | 130,7285 | 0,364444 | 25282,79 | 9,21416 | 7,837059 | 3,414088 | 0,435634 |
| 138,318 | 125,4349 | 0,391 | 14829,73 | 5,798429 | 7,447587 | 3,706183 | 0,497635 |
| 132,6202 | 120,0515 | 0,376663 | 18120,53 | 6,825326 | 7,872875 | 3,770053 | 0,478866 |
| 154,7007 | 140,4723 | 0,416998 | 24784,52 | 10,33509 | 7,866401 | 3,661803 | 0,465499 |
| 140,4547 | 127,493 | 0,42665 | 21715,51 | 9,264922 | 3,708112 | 2,199671 | 0,593205 |
| 134,755 | 121,4587 | 0,359063 | 15685,08 | 5,63194 | 8,807146 | 3,467582 | 0,393724 |
| 141,8918 | 127,0374 | 0,337831 | 12938,85 | 4,37115 | 4,297197 | 6,544048 | 1,522864 |
| 142,5334 | 128,5006 | 0,390404 | 15116,3 | 5,90147 | 4,151179 | 6,125005 | 1,475486 |
| 137,8833 | 123,9879 | 0,34439 | 15467,51 | 5,326853 | 3,715106 | 6,409708 | 1,72531 |
| 150,4657 | 134,3719 | 0,333951 | 11294,59 | 3,771841 | 3,289519 | 5,442413 | 1,654471 |
| 140,928 | 126,5733 | 0,367239 | 11400,7 | 4,186785 | 3,489809 | 7,273822 | 2,084304 |
| 184,1717 | 166,3615 | 0,398926 | 14502,6 | 5,785466 | 3,646869 | 7,865456 | 2,156769 |
| 152,6474 | 137,4825 | 0,405067 | 12422,11 | 5,031783 | 4,444048 | 6,301756 | 1,418022 |
| 156,6323 | 140,6267 | 0,356918 | 12995,56 | 4,638353 | 3,547126 | 6,412575 | 1,807823 |
| 152,4781 | 136,7797 | 0,333813 | 16963,49 | 5,662628 | 3,557343 | 8,081305 | 2,271725 |
| 144,9355 | 130,2455 | 0,362486 | 14874,26 | 5,391705 | 3,782336 | 6,553079 | 1,732548 |
| 145,9295 | 131,5055 | 0,387976 | 14093,18 | 5,467821 | 3,78071 | 6,454606 | 1,707247 |
| 135,231 | 123,6826 | 0,560369 | 11304,24 | 6,334543 | 6,075794 | 5,092625 | 0,838183 |
| 134,001 | 122,043 | 0,448003 | 10840,58 | 4,856611 | 7,371858 | 4,877159 | 0,661592 |
| 133,87 | 121,7659 | 0,429928 | 11775,1 | 5,062445 | 5,830101 | 5,037685 | 0,864082 |
| 131,5906 | 120,2877 | 0,523482 | 5985,417 | 3,133258 | 6,809562 | 4,842868 | 0,711186 |
| 127,4085 | 116,0615 | 0,461855 | 7338,861 | 3,389487 | 6,024333 | 4,739787 | 0,786774 |
| 128,7788 | 117,2154 | 0,429119 | 5272,802 | 2,26266 | 6,449967 | 5,016138 | 0,7777 |
| 143,3498 | 129,913 | 0,365539 | 12677,58 | 4,634151 | 7,22917 | 4,096621 | 0,566679 |
| 134,606 | 121,3614 | 0,334888 | 15889,94 | 5,321346 | 7,895266 | 4,621627 | 0,585367 |
| 147,4948 | 132,0755 | 0,295562 | 22166,05 | 6,551436 | 5,012984 | 4,301257 | 0,858023 |
| 135,8585 | 122,803 | 0,365492 | 9206,515 | 3,364907 | 6,824588 | 4,304725 | 0,630767 |
| 148,3815 | 134,9788 | 0,425758 | 12826,24 | 5,460868 | 6,825472 | 6,031671 | 0,8837 |
| 143,7163 | 129,9626 | 0,393555 | 12383,87 | 4,873727 | 6,533225 | 5,40597 | 0,827458 |
| 141,02 | 128,5742 | 0,507207 | 7217,353 | 3,66069 | 5,745456 | 6,332077 | 1,102102 |
| 132,7978 | 120,7003 | 0,46947 | 6611,404 | 3,103853 | 5,970773 | 5,909134 | 0,989677 |
| 136,753 | 121,4555 | 0,276503 | 9138,724 | 2,526886 | 6,38462 | 3,741295 | 0,585986 |
| 139,19 | 124,1829 | 0,289314 | 8218,935 | 2,377857 | 6,639245 | 4,667012 | 0,702943 |
| 139,6878 | 125,028 | 0,321565 | 9166,94 | 2,947766 | 5,52701 | 4,75775 | 0,860818 |
| 140,6985 | 126,3105 | 0,349274 | 8777,795 | 3,065857 | 5,655803 | 4,569006 | 0,807844 |
| 137,3448 | 123,3525 | 0,346902 | 9625,978 | 3,33927 | 4,677275 | 5,121108 | 1,094891 |
| 135,2823 | 121,1814 | 0,335099 | 6908,433 | 2,315006 | 3,928015 | 4,971807 | 1,26573 |
| 138,7675 | 124,0332 | 0,320178 | 8604,535 | 2,754986 | 5,709101 | 5,33036 | 0,93366 |
| 136,8615 | 121,8451 | 0,291612 | 7843,748 | 2,287327 | 5,716457 | 4,426569 | 0,774355 |

| | | | | | | | |
|---|---|---|---|---|---|---|---|
| 131,9513 | 117,6371 | 0,289766 | 10173,33 | 2,947883 | 6,372532 | 7,514931 | 1,179269 |
| 135,7079 | 121,8314 | 0,330345 | 10588,72 | 3,497927 | 6,026419 | 3,960351 | 0,657165 |
| 133,883 | 119,9897 | 0,315064 | 9749,035 | 3,071571 | 6,592671 | 4,153754 | 0,630056 |
| 133,1508 | 119,8297 | 0,366691 | 14218,4 | 5,213755 | 6,447084 | 3,952951 | 0,613138 |
| 134,7589 | 120,9941 | 0,333009 | 14235,03 | 4,74039 | 5,544654 | 3,567291 | 0,643375 |
| 138,7056 | 122,9489 | 0,275587 | 12324,61 | 3,396501 | 5,964285 | 4,372276 | 0,733076 |
| 139,3568 | 125,7033 | 0,333951 | 8999,175 | 3,005283 | 4,079228 | 3,19853 | 0,784102 |
| 134,2957 | 119,8102 | 0,282496 | 9825,791 | 2,775745 | 5,121023 | 5,013534 | 0,97901 |
| 138,2059 | 123,1799 | 0,285231 | 8655,89 | 2,468925 | 5,106692 | 5,009486 | 0,980965 |
| 135,7288 | 123,2463 | 0,430673 | 13241,4 | 5,70272 | 4,922651 | 3,40317 | 0,691329 |
| 128,6739 | 115,5804 | 0,345441 | 7003,037 | 2,419138 | 6,368018 | 4,512024 | 0,708544 |
